# Multimodal frailty detection in primary care using a portable sensor-based platform: exploratory results

**DOI:** 10.3389/fragi.2026.1695517

**Published:** 2026-04-02

**Authors:** Florian Legrand, Ioannis Bargiotas, Matthieu Ndumbi Lukuenya, Jean-Marc Eychene, Evelyne Alastor, Lise Haddouk, Christophe Labourdette, Sébastien Leruste, Jean-Marc Franco, Frédéric Sandron, Pierre-Paul Vidal

**Affiliations:** 1 Institut de Recherche pour le Développement Ceped—UMR 196, Université Paris Cité, Paris, France; 2 UFR Santé, University of La Réunion, Saint-Pierre, France; 3 Centre Borelli, UMR 9010, Centre National de la Recherche Scientifique, ENS Paris-Saclay, Université Paris-Saclay, Paris, France; 4 CHU site Sud Saint Joseph, Saint-Joseph, France; 5 INSERM CIC 1410, CHU de La Réunion, Saint-Pierre, France

**Keywords:** postural balance, digital health, fall risk, frailty, gait analysis, ICOPE, primary care

## Abstract

**Introduction:**

Frailty reflects age-related decline across multiple physiological systems, reducing resilience and increasing risks of falls, hospitalization, disability, and mortality. Scalable approaches are needed to identify pre-frailty earlier in community-dwelling older adults and enable timely prevention in primary care.

**Objective:**

To develop and evaluate a multivariable sensor-based framework for early frailty detection using standardized gait and balance assessments in general practice.

**Methods:**

We conducted a prospective cohort study (2021–2024) in southern Réunion Island among retired adults aged ≥65 years recruited in primary care. The protocol included: (1) baseline general practitioner (GP) assessment with expert frailty rating, Fried phenotype, and WHO ICOPE Step 1; (2) telephone assessment of mental health, self-rated health, and quality of life; (3) outpatient instrumented evaluation combining IMU-based gait analysis, force-platform posturography, grip strength, and ICOPE Step 2 measures; (4) monthly falls surveillance over 6 months; and (5) repeat instrumented gait and balance assessment at 6 months. Correlation analyses and machine-learning models examined relationships between frailty measures and the discriminative value of sensor-derived and multimodal predictors.

**Results:**

Among 145 participants (mean age 71 ± 5 years), 98.5% had impairment in at least one intrinsic capacity domain at baseline, most commonly vision (77.7%), locomotion (53.1%), hearing (52.5%), and psychological (27.3%). Sedentary behavior was frequent (77%). Expert frailty scores correlated with the Fried phenotype, whereas associations with self-rated health were weaker. Models based on sensor parameters alone showed limited ability to reproduce Fried-defined frailty, while multimodal models integrating clinical and questionnaire variables improved discrimination. Over 6 months, kinesiotherapy and regular physical activity were associated with improved postural control metrics (including center-of-pressure features and mediolateral sway), while changes in gait speed were modest.

**Conclusion:**

An IoT-supported platform combining quantitative gait, balance, and grip strength measures with targeted questionnaires is feasible in outpatient primary care and yields frailty estimates broadly consistent with GP assessment. However, subjective and clinical inputs remain essential to capture psychological aspects of frailty not fully reflected by sensor signals alone. These findings support scalable frailty screening and longitudinal monitoring, and warrant validation in larger samples, including deployment by trained non-medical personnel and integration into precision-prevention pathways.

## Highlights

### Technological innovation

- Developed a portable, sensor-based platform combining IMU gait analysis, force-plate balance assessment, grip strength, and standardized questionnaires for early frailty screening in primary care.
- Designed to support scalable assessments compatible with non-specialist workflows.

### Clinical relevance and diagnostic coherence

- Platform outputs showed coherent associations with clinician-observed instability and established frailty screening measures.
- Multimodal models integrating sensor-derived and clinical/questionnaire variables improved frailty discrimination compared with sensor data alone.

### Participatory and multidomain assessment

- Combined clinician-rated items and patient-reported outcomes to complement objective functional measures.
- Enables a broader multidimensional characterization of vulnerability beyond physical performance alone.

### Scalability and prevention potential

- Provides a time-efficient and potentially cost-effective approach to support proactive identification of early vulnerability in community-dwelling older adults.
- Supports longitudinal monitoring strategies aimed at preventing functional decline.

### Future perspectives

- Sets the groundwork for individualized longitudinal monitoring and the future development of digital twin–inspired approaches for personalized prevention pathways.
- Future work will extend validation to larger cohorts and longer follow-up, including fall-related outcomes and broader psychosocial determinant

## 1 Introduction

In 2024, approximately 10% of the global population was aged 65 years or older, and this proportion is projected to approach one-quarter by 2,100, with a parallel increase in the number of older adults living with chronic conditions and functional limitations (e.g., Parkinson’s disease, dementia, stroke, falls, or reduced functional capacity) (United Nations Department of Economic and Social Affairs and Population Division, 2025; Eurostat, 2023; Fried L. P. et al., 2001; Sirven and Bourgueil, 2016; Romero-Ortuno, 2011). This demographic transition is already associated with increasing pressure on caregivers and health systems, as more individuals experience progressive loss of independence and require long-term support (De Na et al., 2016; Fried T. R. et al., 2001).

Preventing loss of autonomy has therefore become a central objective for clinicians, researchers, and policymakers, with an emphasis on primary and secondary prevention, health promotion, and the preservation of functional capacity within integrated care pathways (World Health Organization, 2023; Béland et al., 2006). A key element of these strategies is the timely identification of frailty (Rollan et al., 2011), defined as a clinical state of decreased physiological reserve and diminished resilience to stress that substantially increases the risk of hospitalization, dependency, and institutionalization (Fried L. P. et al., 2001; Romero-Ortuno, 2011). Within this continuum, pre-frailty represents a particularly important and potentially reversible stage that precedes largely irreversible loss of autonomy and offers a specific window for intervention (Fried L. P. et al., 2001; Gill et al., 2006; Colvez et al., 2003).

Implementing this preventive approach in practice requires systematic, community-level screening from midlife onward, early detection of pre-frailty, and restoration of physiological robustness through targeted, context-appropriate interventions. Existing tools—ranging from self-report questionnaires (WHO, 2024a; Cesari et al., 2014), to clinical identification by primary care physicians and primary-care screening instruments (Rougé Bugat et al., 2012; Tavassoli et al., 2014; Haute Autorité de santé, 2013), to standardized geriatric assessments (Stuck et al., 1993)—have contributed to this objective but present several limitations. Self-reporting has limited capacity to detect subtle or asymptomatic changes that patients may not perceive, and primary-care screening tools for frailty and intrinsic capacity lack sufficient validation to discriminate between frailty stages or specific domains such as locomotion, cognition, psychological status, vitality, hearing, and vision (Haute Autorité de santé, 2013; Audiffren et al., 2016). The Comprehensive Geriatric Assessment (CGA), which provides a detailed medico-social evaluation followed by a personalized care plan, has demonstrated effectiveness in preventing loss of autonomy (Stuck et al., 1993), but it is time-consuming and requires specialized geriatric expertise usually confined to tertiary care settings, thereby limiting its accessibility and scalability (Windak et al., 2024; Legrand et al., 2021a). In parallel, wearable sensor technologies and machine learning enable continuous and objective assessment of functional, behavioral, and mobility-related parameters associated with frailty trajectories in real-world settings. However, most existing digital approaches remain limited to single-domain indicators, do not achieve sufficient detection performance—particularly in terms of area under the curve (AUC) and specificity—and are primarily evaluated in controlled, hospital-based environments, thereby limiting their transferability to routine ambulatory care and community-dwelling populations (Schwenk et al., 2015; Apsega et al., 2020).

In France, the rollout of the ICOPE prevention program, targeting community-dwelling adults aged 60 years and older and currently being scaled at the national level, further illustrates that face-to-face assessments alone are insufficient to reach large numbers of older adults in a timely and repeated manner. To operationalize ICOPE’s multi-step pathway at scale—initial self-screening, confirmation, personalized care planning, and follow-up—remote monitoring platforms are needed to support continuous reporting of intrinsic capacity, automated alerts to professionals, and coordination across territorial care networks (Tavassoli et al., 2022). SUNFRAIL+ is an ICT-supported, multidimensional program that links the brief 9-item SUNFRAIL tool to in-depth bio-psycho-social assessments in community-dwelling older adults, enabling comprehensive frailty profiling beyond single-domain measures (De Luca et al., 2025). Its cascade model, in which positive screening items trigger domain-specific questionnaires and physical tests, supports risk stratification and tailored preventive interventions rather than generic recommendations, but also entails additional time, training, and coordination, thereby increasing workload and organizational complexity for participating services. Beyond functional decline, frailty is increasingly understood as a multidimensional condition involving sensory, neurophysiological, and inflammatory mechanisms. Recent evidence suggests that quantitative sensory and physiological markers can contribute to the prediction of functional outcomes, supporting the broader use of digital biomarkers in aging research (Murphy et al., 2025). However, monitoring these biomarkers requires specialized personnel and resources, which could make mass prevention difficult. Finaly, field-based prevention workshops often attract individuals who are already robust, thereby limiting their reach among those at higher risk. In La Réunion, the *Atout Âge* workshops—implemented as part of the national “Ageing Well” prevention program delivered by French pension funds, and deployed locally before the introduction of ICOPE—illustrate this limitation: although designed to promote healthy ageing, they predominantly reached healthier and more autonomous older adults, highlighting the challenge of engaging frailer populations (Legrand et al., 2021a; Annika, 2024; Direction de la Recherche Études and de l’Évaluation et des Statistiques, 2020). Overall, current prevention pathways remain difficult to implement and sustain at the population level.

These limitations suggest that frailty detection should extend beyond clinical and hospital settings. Demedicalization is important not only for scalability, but also for supporting autonomy, dignity, and equity of access by enabling older adults to be assessed in familiar, non-stigmatizing environments and outside specialist services (Fernández-Carnero et al., 2024). In this context, digital health solutions have emerged as promising tools to support scalable, continuous, and context-aware monitoring of older adults in their daily environments. Recent studies indicate that sensor-based systems, mobile applications, and machine-learning algorithms can be used to screen for frailty and pre-frailty in community-dwelling older adults, for example, through gait analysis from wearable or ambient sensors, passive monitoring of activities of daily living, or integration of electronic health record data into predictive models (Vidal et al., 2020; Michel, 2024). Such approaches may enable earlier identification of subtle functional declines, reduce dependence on in-person clinical contacts, and be deployed in primary care, senior centers, or at home, thereby expanding the reach of prevention programs. Digital platforms can also facilitate personalized feedback and remote follow-up, which are important for maintaining engagement and supporting long-term prevention trajectories in aging populations (Czaja, 2017; Wilson et al., 2021).

This study proposes a concrete step toward demedicalizing frailty detection by presenting a validation of a digital frailty-detection platform. The system combines wearable inertial measurement units (IMUs), force-plate balance assessment, grip strength measurement using a strain gauge, and standardized questionnaires to support longitudinal frailty monitoring.

The objective of the 5P Echelle cohort study presented here was to develop and assess the performance of an advanced sensor-based multivariable platform for the detection of early frailty signals in older adults, using standardized gait and balance recordings collected in an outpatient primary care setting. The assessment was performed with a portable platform integrating IMU-based gait analysis, static balance evaluation, grip strength measurement, and standardized questionnaires. This study reports the baseline results of the 5P-Échelle cohort and examines the cross-sectional and longitudinal discriminatory ability of sensor-derived parameters for frailty status in primary care. Exploratory analyses assessed whether sensor-based evaluations could identify pre-frailty and frailty using established reference outcomes, and aimed to identify additional gait- and balance-related parameters, as well as clinical and self-reported questionnaires, to improve detection accuracy or identify the most discriminative parameters.

## 2 Materials and methods

### 2.1 Study design, setting and participants

We conducted a prospective cohort study to validate an IoT-based frailty detection system from 05/10/2021 to 03/01/2024. Participants were retired people over 65 years of age affiliated to a social security scheme, recruited by consecutive sampling from patients consulting the practices of 11 general practitioners in the south of Reunion, France. All participants were recruited by GPs trained for the study, who checked the inclusion and exclusion criteria and obtained written informed consent. Exclusion criteria included people covered by Articles L1121-5 to L1121-8 of the French Public Health Code—i.e., ‘protected persons’ such as adults under legal protection, individuals deprived of liberty, or those unable to provide informed consent—as well as participants who did not understand French.

**FIGURE 1:**
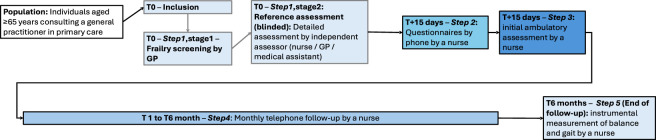
Study timeline and assessment procedure.

Step 1. After inclusion of the participant by the general practitioner, the latter performed a hetero-assessment of frailty. A trained assessor—who could be a medical assistant, a nurse, or an investigator physician specifically trained for the study—then carried out the tests of the initial medical visit, including screening tests and the establishment of the frailty profile according to Fried’s criteria. These tests were performed in a separate room and were blinded to the GP’s evaluation.
Step 2. Within 15 days, the study coordinating nurse contacted participants to complete questionnaires by telephone and to schedule an in-depth outpatient clinical assessment.
Step 3. This assessment, performed by the same nurse, included the baseline functional capacity tests.
Step 4. Participants were then followed monthly by telephone for 6 months by the coordinating nurse to monitor the occurrence of falls, in accordance with the methodological recommendations of the Prevention of Falls Network Europe (ProFaNE) (Lamb et al., 2005; Romli et al., 2021).
Step 5. A final visit was conducted at 6 months, also by this nurse, to record gait and balance. The study design and timeline of assessments are presented in Figure 1.

Participants met the general practitioner and the assessors in separate rooms. The results of each step were documented in separate case report forms linked through a unique participant code. Participants were instructed not to share results from previous assessments in order to maintain blinding. Blinded assessors did not have access to the GP’s clinical evaluations, and the coordinating nurse did not have access to the results of the tests performed by either the general practitioner or the blind assessor. All investigators were trained in standardized training sessions to ensure consistent administration of clinical tests and sensor-based tasks. In the event of abnormal test findings, the participant was informed and, with the participant’s agreement, the usual general practitioner was notified to facilitate appropriate medical evaluation and follow-up care.

**FIGURE 2:**
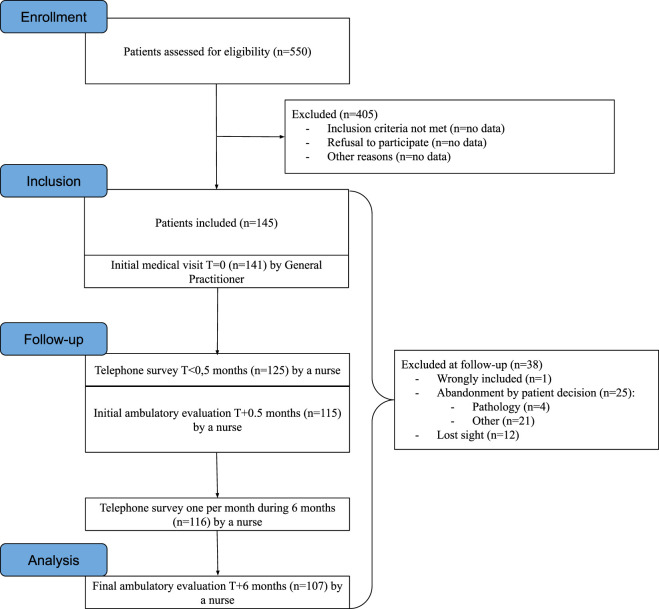
Flow chart of the 5PE study.

One hundred and forty-five patients were included, 141 of whom completed the initial medical examination. The participation rate in this study was 26%. One hundred and seven patients (74%) completed the 6-month follow-up and evaluation. Of the 38 patients who did not complete the study, 26 dropped out at the patient’s own decision: 4 because of pathologies and 21 for other reasons. Twelve patients were lost to follow-up. One participant was wrongly included. These data are presented in the flow chart Figure 2.

This study was approved by the Research Ethics Committee of “Université Paris-Cité” N° 2020-05-EYCHENE-VIDAL. The protocol was updated following the outbreak of COVID-19 (Legrand et al., 2021b). Clinicaltrials.gov ID No.: NCT05090241. At the inclusion visit, oral and written information was given by the investigator on the 5P ECHELLE study. The collection of consent was recorded in the visit report. All adverse events were recorded. All data were stored and processed in full compliance with the General Data Protection Regulation (GDPR). The certified health-data cloud used for this study was hosted in France. This study was declared to the CNIL under registration number 2222781. Death and any other reasons for premature interruption of follow-up were recorded during the follow-up and reported on a special form. Funders had no influence on the design, conduct, analysis, interpretation and writing of the study.

### 2.2 Measurement and outcomes

Our study involved five steps.

#### 2.2.1 First step: Information gathering at baseline

Information gathered at baseline by a General Practitioner (GP) and an assessor following inclusion during an initial visit.

This first visit took place in two stages:

First stage, a clinician-rated frailty score was obtained from the patient’s general practitioner using a single global question: “Based on your clinical judgment, how would you rate this patient’s overall health status on a scale from 0 (very poor) to 10 (excellent) (Vernerey et al., 2016)?”

Second stage, a detailed assessments conducted by a trained blind assessor in a separate room. The following information were collected:

- Integrated care for older people (ICOPE) Step 1 as defined by WHO (WHO, 2024b)
- Score CODEX (normal or abnormal) (Belmin et al., 2007)
- Clinical data: sex, height, weight, Body-mass index (kg/m^2^), previous weight (at 12 months).
- The Falls risk for older people in the community (FROP-COM) (Russell et al., 2008)
- Fall history in last 12 months (Yes or no and numbers of falls). According to the WHO, a fall is defined as an event in which an individual inadvertently falls to the ground or any other surface below his/her level of origin.
- Short Physical Performance Battery (SPPB) (score: 0–12, with lower scores indicative of worse locomotion) with 4 m gait time (Guralnik et al., 1994)
- Grip strength (Fried L. P. et al., 2001)
- Instrumental measurement of balance (Legrand et al., 2021a)

The information collected during these two stages served to implement the frailty phenotype proposed by Fried and al (Fried L. P. et al., 2001). It was used in the present study as reference measure of frailty. The frailty phenotype has shown to be predictive of major health related negative outcomes in older persons (Fried L. P. et al., 2001; Romero-Ortuno, 2011).

The Fried criteria are a list of five criteria.

- Involuntary weight loss: measurement of current weight minus 12-month weight from medical record. If > 4.5* *kg then the older adult is frail according to the weight loss criterion.
- Exhaustion: Assessed through the question: *“Over the past 4 weeks,* how *often* have *you felt exhausted?”* with response options: all the time (United Nations Department of Economic and Social Affairs and Population Division, 2025), very often (Eurostat, 2023), often (Fried L. P. et al., 2001), a few times (Sirven and Bourgueil, 2016), rarely (Romero-Ortuno, 2011), or never (De Na et al., 2016). Participants responding “rarely” or “never” were classified as frail on this criterion.
- Gait speed: Measured by timing a 4-m walk from a standing start. Classification of frailty was based on established cut-off values proposed by Fried L. P. et al. (2001).
- Grip strength: Assessed using a digital dynamometer and evaluated against Fried et al.‘s sex- and BMI-adjusted thresholds to determine frailty status Fried L. P. et al. (2001).
- Physical activity: Evaluated *via* the question: “What is your level of physical activity*?”* Responses included: “regular physical activity (at least 2–4 h per week)” (scored as non-frail) and “none or mainly sedentary” (scored as frail).

A person is frail according to the Fried score, if they meet three of the five criteria, pre-frail if they meet one or two criteria, or robust if they do not meet any criteria.

In the current study, the physical activity criterion was not assessed using the Minnesota Leisure Time Physical Activity Questionnaire (Richardson et al., 1994), due to its length and complexity, which limit its feasibility in a primary care context. Instead, a simplified and previously validated assessment method was employed (Cherubini et al., 2015; Patel et al., 2006; Cesari et al., 2015), which has shown substantial agreement with the original questionnaire (agreement rate = 77.8%, κ = 0.537; *p* < 0.001) (Cesari et al., 2014). Fried’s criteria were collected during this evaluation, but the results were not communicated to the assessor.

Balance was measured on a force platform. The older adult was placed barefoot on the force platform. The measurement was performed for 30 s with the eyes open, then with the eyes closed for 30 s. Postural control acquisition data, and more specifically the displacement of the center of pressure, were measured in the medio-lateral and antero-posterior dimensions.

#### 2.2.2 Second step, an additional assessment by phone

Once the initial medical examination had been carried out, a telephone call was made by a nurse with the following assessment.

- *Assessment of Deprivation and Health Inequalities in Health Examination Centers (EPICES)* score (Labbé et al., 2007)
- Standardized Assessment of Personality - Abbreviated Scale (SAPAS) (Moran et al., 2003),
- Participant self-rated frailty/overall health (Auto_Evaluation). Participants were asked to reflect on the past week, including today: *“Thinking about how you have felt over the past week (including today), how would you rate your overall state of health on a scale from 0 to 10, where 0 = as bad as possible and 10 = as good as possible?“,*
- FRAGIRE Grid (Vernerey et al., 2016). It was developed by the Interregional Gerontology Center of *Bourgogne–Franche-Comté* and includes one administrative section; three cognitive and functional tests (Cued Recall Memory Score, Isaacs Set Test, and gait speed—the latter not performed during telephone assessments); 17 questions covering perceived health status, physiological symptoms, depression, pleasure, suicidal ideation, social and cultural environment, caregiving role, sexuality, oral health, and mobility; and one question for the assessor regarding their overall perception of the participant’s health status.“,
- Medical Outcomes Study 36-item Short-Form-36 (SF-36) (Ware and Sherbourne, 1992),
- Modified falls efficacity scale (MFES) (Perrot et al., 2018),
- Hospital anxiety and depression scale (HADS) (Zigmond and Snaith, 1983; Bocéréan and Dupret, 2014).

#### 2.2.3 Third step, an initial ambulatory assessment

Within 15 days, a nurse trained in the study was to carry out an initial ambulatory assessment.

- Instrumental gait measurement.
- ICOPE Step 2
  ○ Hearing Handicap Inventory for the Elderly–Screening (HHIE-S) (score: 0–40, with higher scores indicative of worse hearing ability) (Servidoni and Conterno, 2018)
  ○ Measurement of WHO visual acuity using the Raskin E scale (deficit: yes or no) (Integrated care for older peopleb)
  ○ Mini mental state examination (MMSE) (scores: 0–30, with lower scores indicative of worse cognitive impairment)
  ○ Mini Nutritional Assessment (MNA) (score: 0–30, with higher scores indicative of better nutrition) (Guigoz et al., 1996)

The gait test was carried out using sensors that analyze and retrieve data relating to the participant’s gait (gait speed, step length, duration of the double stance phase). Four Xsens® wireless inertial measurement unit (IMU) motion tracker sensors were positioned on the clothing using headbands as follows: one at forehead level on the median sagittal line, the second opposite the L4 vertebra and the last two on the dorsal surface of the right and left feet at mid-foot level. The patient then walked 10 m in a straight line, turned around and walked another 10 m. IMU sensors and the force platform (Nintendo® Wii Board Balance force platform) were calibrated according to manufacturer guidelines.

#### 2.2.4 Fourth step, for 6 months, monthly monitoring for fall

The participant was then monitored monthly during 6 months for falls, in accordance with the guidelines of the Prevention of Falls Network Europe (ProFaNE) (Lamb et al., 2005).

#### 2.2.5 Fifth step, information at 6 months by a nurse

At 6 months, the final ambulatory evaluation was scheduled, with instrumental measurement of balance and gait.

The index test performers and the reference standard assessors were blind to the results of the reference and index tests respectively.

### 2.3 “Digital technologies and data integration”

Digital assessments were conducted using the portable SmartCheck® platform, an IoT-based frailty screening system developed by the Borelli Center at the University of Paris Cité. The platform integrates three complementary measurement technologies: (United Nations Department of Economic and Social Affairs and Population Division, 2025): wearable inertial measurement units (IMUs) for standardized gait analysis, (Eurostat, 2023), a portable force platform for static balance assessment, and (Fried L. P. et al., 2001) a digital strain-gauge handgrip dynamometer. The system consists of a touch-sensitive tablet connected to gait sensors (Xsens®), a balance force platform (Nintendo® Wii Balance Board), and a secure server performing automated signal processing and mathematical analyses (Audiffren et al., 2016; Bargiotas et al., 2018; Audiffren and Contal, 2016).

Static balance assessment was performed using the force platform. Participants stood barefoot on the platform, arms alongside the body, with feet slightly apart (not exceeding shoulder width). They were instructed to remain as still as possible during the recording. Postural control was assessed over two consecutive 25-s trials: first with eyes open, then with eyes closed. Center of pressure (CoP) displacement—reflecting the postural control mechanisms involved in maintaining balance—was recorded in the medio-lateral and antero-posterior directions (Ba et al., 2002; Winter et al., 1998). Data were acquired *via* a dedicated Windows® Smart Check® application developed by the Borelli Center and displayed as statokinesiograms (Audiffren et al., 2016; Audiffren and Contal, 2016). The balance outcomes retained for analysis were the mean CoP velocity (cm/s), medio-lateral variability (ML, cm), defined as the standard deviation of CoP displacement, and micro-falls (cm), defined as the maximum CoP displacement observed over any 1-s interval during the recording (Ba et al., 2002).

Gait assessment was performed using wearable IMUs to capture spatiotemporal walking parameters, including gait speed, step length, and double support duration. Four Xsens® MTw sensors (Xsens® Technologies) were attached over clothing using elastic straps: one on the forehead along the sagittal midline, one over the L4 vertebra, and two on the dorsal surface of each foot at midfoot level. Prior to testing, participant anthropometric data were entered for calibration, followed by a brief static standing phase to synchronize the sensors with the tablet. Participants then walked 10 m at their usual pace, performed a 180° turn, and walked back 10 m. IMU-derived average gait speed (AvgSpeed), computed from sensor-based measurements, was used exclusively as an independent predictor in supervised models and did not contribute to the definition of the reference frailty outcome.

### 2.4 Statistical analysis

All data were collected using an electronic case report form (eCRF). Raw data from the IMU sensors and the force platform were automatically collected *via* a secure cloud-based platform and securely linked to each participant’s unique study code. Continuous variables with Gaussian distribution are presented as mean ± standard deviation (SD). Categorical variables are reported as absolute numbers and percentage frequencies. Statistical comparisons of means were conducted using the Wilcoxon signed**-**rank test, while proportions were compared using the chi-square test. When applicable, a Bonferroni correction was applied to adjust for multiple comparisons.

Results obtained from various sources including the hetero-assessment (completed by the general practitioner), the initial medical visit (performed by a research-trained assessor), telephone-based evaluations, and the ambulatory visit were compared under blinded conditions. Correlation and concordance analyses were performed to assess the level of agreement across raters and assessment methods. A *p*-value of <0.05 was considered statistically significant.

The primary outcome was the convergent validity between digital assessments and clinical frailty measures. Secondary outcomes included the discriminative ability of digital markers across robustness, pre-frailty, and frailty levels, and their predictive validity.

Frailty scores used in the study were defined as follows for statistical analysis.

○ Frailty_Score (continuous*, range:* 0*–*10*;* 10 = best*)*: A global frailty score assigned by the expert prior to the other evaluations and derived from the validated FRAGIRE grid.
○ Frailty_State (binary): Frailty status determined by the general practitioner immediately following the Frailty_Score assessment.
○ Fried_Score (continuous*, range:* 0*–*5*;* 5 = worst*)*: A composite score based on Fried’s frailty criteria.
○ Fried_State (binary): Frailty classification based on the Fried_Score; individuals scoring ≥3 were considered frail.
○ Auto_Evaluation (continuous*, range:* 0*–*10*)*: Self-assessed frailty score reported by the participant using the FRAGIRE grid.
○ Falls (binary at M0 and M6)**:** Self-reported fall occurrences at baseline (M0) and at the 6-month follow-up (M6).

Three categories of statistical tests were conducted based on the variable types being compared.

1. Continuous vs. Continuous Variables: Spearman’s rank correlation coefficient (rho) was used to assess relationships between continuous frailty measures, as it does not assume normality and is appropriate for ordinal scales. Correlation strength was interpreted as: weak (<0.3), moderate (0.3–0.5), and strong (>0.5).
2. Binary vs. Binary Variables: Chi-square tests were performed to examine associations between binary frailty classifications, with Cramer’s V calculated as a measure of effect size. Effect sizes were interpreted as: negligible (<0.1), weak (0.1–0.2), moderate (0.2–0.3), and strong (>0.3).
3. Binary vs. Continuous Variables: Mann-Whitney U tests were used to compare continuous frailty measures between binary groups (e.g., frail vs. non-frail), with effect size r calculated as |Z|/√N. Effect sizes were interpreted as: negligible (<0.1), small (0.1–0.3), medium (0.3–0.5), and large (>0.5).

Statistical significance was initially set at p < 0.05, with additional thresholds at p < 0.01 and p < 0.001 indicated for stronger significance levels. Given the multiple statistical tests performed (15 tests in total), we also applied Bonferroni correction to control for family-wise error rate, resulting in an adjusted significance threshold of p < 0.00333 (0.05/15). A color-coded heat map visualization was created to clearly display which relationships remained significant after Bonferroni correction, with exact p-values provided for all variable pairs.

The dataset contained missing values in key features. To address this issue, we just excluded the incomplete cases from our analysis, ensuring that the remaining dataset maintained its integrity and allowed for accurate interpretation of the results.

#### 2.4.1 Machine learning framework

We performed a rigorous machine learning benchmarking choosing algorithms that show balance between predictive performance and interpretability. Four tree-based ensemble methods—Random Forest, XGBoost, CatBoost, and LightGBM—were evaluated due to their proven effectiveness on structured healthcare data.

These methods are widely known for their robustness and resistance to overfitting, their general ability to handle high-dimensional and missing data, and the advanced interpretability tools (such as feature importance and out-of-bag error estimation). Moreover, boosting algorithms (such as XGBoost) were included for sequential gradient boosting framework and advanced regularization functionalities. Finally, algorithms such as LightGBM were employed for its computational efficiency on large, high-dimensional datasets.

Hyperparameter optimization was performed implementing a Bayesian optimization with a Tree-structured Parzen Estimator (TPE) to efficiently explore the hyperparameter space. TPE was preferred over Gaussian Processes because it also naturally handles categorical and discrete hyperparameters and offers faster optimization without costly matrix inversions.

We intentionally decreased the complexity of the models performing binary classifications for Frailty_State (binary) instead of regression or multiclass ordinal classifications. Model performance was optimized for ROC-AUC using stratified 5-fold cross-validation, with 25 optimization trials per model.

The four methodologies were inserted in the same validation pipeline followed well-established approaches where dataset were split into training (80%) and testing (20%) sets using stratification to preserve class distributions, Missing values were imputed using cross-validated KNN with feature-aware distance metrics, while class imbalance was addressed *via* combination of Synthetic Minority Oversampling Technique (SMOTE) and TOMEK Links (SMOTETomek) to synthesize minority samples. Class imbalance and imputation methodology was addressed exclusively during training, while validation and test sets remained unchanged (except of the application of imputation). Final model evaluation used ROC-AUC to finally assess the predictive performance of the models.

Our approach is consistent with recent multicenter efforts aiming to structure frailty detection using machine-learning models across heterogeneous clinical settings (Fernández-Carnero et al., 2025).

## 3 Results

### 3.1 Clinical characteristics of the population and initial medical visit

The baseline characteristics of the 145 participants are presented in Table 1. The mean age of the population was 71 ± 5 years, with a male-to-female ratio of 1.4:1. The general practitioners’ initial assessment of the patients’ overall health scored an average of 7.5 ± 1.6.

**TABLE 1:** Clinical characteristics of the population at the initial medical visit.

Characteristics	Population (n = 139)
	Average ± SD or n (%)
Age (years)	71 ± 5
Sex (male)	85 (59.1)
Person’s overall state of health, hetero assessment by GP (/10) n = 144	7.5 ± 1.6
Person’s overall state of health, hetero assessment by GP (Yes/no) n = 144	41 (28.5)
ICOPE Step 1	
Vision problems	108 (77.7)
Ophthalmologist consultation <2 years	103 (74.1)
Locomotion	77 (53.1)
Hearing - Whispered Voice Test (Abnormal)	73 (52.5)
Audiometry or ENT consultation within the last 2 years	35 (25.2)
Psychological problems	38 (27.3)
Nutrition	32 (23)
Memory or orientation problems	31 (22.3)
Score CODEX (Abnormal)	43 (30.9)
FROP-COM SCREEN (/9)	1.1 ± 1.5
Low risk of falls (≤3)	128 (82.8)
High risk of falls (4–9)	10 (7.2)
FROP-COM (/60)	9.9 ± 5.3
Mild falls risk (0–11)	90 (65.2)
Moderate falls risk (12–18)	35 (25.4)
High falls risk (19–60)	13 (9.4)
BMI	26.4 ± 4.8
Weight change (kg) 12 months	−0.34 ± 3.1
Level of physical activity	
Regular physical activity (at least 2–4 h a week)	46 (33)
None or mainly sedentary	93 (77)
Adapted physical activity (APA)	14 (10)
Regular treatment by a physiotherapist (Yes)	26 (19)
Grip strength (kg)	
Female	34.7 ± 8.6
Male	19.0 ± 6.1
SPPB (/12)	9.37 ± 1.8

GP, general practitioners; ICOPE, integrated care for older people; ENT, nar, Nose and Throat specialist; CODEX, COgnitive Disorders EXamination; FROP-COM, the falls risk for older people in the community; BMI, body mass index; SPPB, short physical performance balance.

During the ICOPE Step 1 assessment, 127 participants (98.5%) showed potential declines in at least one intrinsic capacity domain. Specific findings included potential vision decline in 108 participants (77.7%), locomotion issues in 77 (53.1%), hearing impairment in 73 (52.5%), psychological issues in 38 (27.3%), vitality decline in 32 (23%), and cognitive issues in 31 (22.3%). Additionally, 76 participants (54.7%) exhibited an abnormal CODEX score, and 13 (9.4%) were classified as having a high risk of falling based on the FROP-COM.

Regarding physical activity, 93 participants (77%) were predominantly sedentary, 14 (10%) engaged in adapted physical activities, and 26 (19%) regularly received physiotherapy. Mean hand grip strength was 34.7 ± 8.6 kg for men and 19.0 ± 6.1 kg for women. The mean Short Physical Performance Battery (SPPB) score was 9.37 ± 1.8. Further details are presented in Table 1.

At the telephone survey, data from 123 participants were analyzed. Vulnerability (EPICE scale) was identified in 74 participants (60.2%). Anxiety and depression (HADS) were present in 31 (25.2%) and 9 (7.3%) participants, respectively.

Based on the FRAGIRE scale, 48 participants (39%) were robust, 66 (54%) were pre-frail, and 8 (7%) were frail. SF-36 scores highlighted impairments across various domains, with physical functioning scoring 77.8 ± 22.5 and general health 59.2 ± 17.6 (Table 2).

**TABLE 2:** Clinical characteristics of the population at the telephone survey.

Characteristics	Population (n = 123)
	Average ± SD or n (%)
MFES	9.4 ± 1.1
EPICE	29.5 ± 17.1
Vulnerability	74 (60.2)
HADS Anxiety (≥11)	31 (25.2)
HADS Depression (≥11)	9 (7.3)
SAPAS positive (≥3)	43 (35)
Person’s overall state of health, Auto-assessment	7.6 ± 2.1
FRAGIRE	42 ± 1
FRAGIRE Robust	48 (39)
FRAGIRE pre-frail	66 (54)
FRAGIRE frail	8 (7)
SF36-Physical functioning	77.8 (22.5)
SF36-Role limitations due to physical health	74.2 (38.3)
SF36-Role limitations due to emotional problems	86.7 (29.5)
SF36-Emotional wellbeing	62.8 (19.7)
SF36-Social functioning	79.8 (21.7)
SF36-Pain	66.6 (25.6)
SF36-General health	59.2 (17.6)

MFES, Modified Falls Efficacy Scale; EPICE, Assessment of Deprivation and Health Inequalities in Health Examination Centers; HADS, Hospital anxiety and depression scale; SAPAS, -Standardised Assessment of Personality - Abbreviated Scale; SF36, Short Form.

In the ambulatory assessment (n = 118), the HHIE-S score averaged 5 ± 7, with 80.5% scoring in the normal range (0–8). The MMSE score averaged 25 ± 4, with 41% scoring below 24. Nutritional status (MNA) was mostly normal, with 88.9% scoring between 12 and 14. Additional details are provided in Tables 3.

**TABLE 3:** Clinical characteristics of the population at the ambulatory assessment.

Characteristics	Population (n = 118)
	Average ± SD or n (%)
HHIE-S Score (/40)	5 ± 7
Score 0–8	95 (80.5)
Score 10–16	10 (8.5)
Score 18–40	13 (11.0)
MMSE Score (/30)	25 ± 4
Number of MMSE score (<24)	48 (41)
MNA screening score (/14)	13 ± 2
12–14 points: normal nutritional status	104 (88.9)
8–11 points: at risk of undernutrition	3 (2.6)
0–7 points: confirmed undernutrition	10 (8.5)
MNA score (/30) n = 13	21.4 ± 4
Adequate nutritional status (^3^ 24)	4 (30)
Risk of malnutrition (≤17 MNA <24)	8 (62)
Protein-calorie malnutrition (<17)	1 (8)
Measurement of WHO visual acuity using the Raskin E scale (Abnormal)	2 (1.7)
Wearing glasses	67 (56.8)

The median time interval between the index test and the reference standard was 13 ± 44 days. Over the course of the study, eight falls involving seven participants (6%) were recorded through monthly telephone follow-ups and diary entries.

The median time interval between the index test and the reference standard was 13 ± 44 days.

Eight falls for seven participants (6%) were recorded in the monthly telephone and diary follow-up.

### 3.2 Detecting frailty and the risk of falls using innovative self-, hetero-evaluative, clinical and individual longitudinal approaches

#### 3.2.1 Descriptive statistics

The analysis included 105 participants with complete data across all measures. Based on expert assessment (Frailty_State), 28 participants (26.7%) were classified as frail, whereas 24 participants (22.9%) were classified as frail using the Fried phenotype definition (Fried_State ≥3). The median Frailty_Score was 8.0 (range: 1–10), and the median Fried_Score was 2.0 (range: 0–5). Instrumented gait and balance measurements at baseline and at the 6-month follow-up are presented in Table 4.

**TABLE 4:** Instrumented gait and balance measurements at baseline and 6-month follow-up.

Domain	Measurement	Baseline n	Baseline mean ± SD	Baseline median [IQR]	Follow-up n	Follow-up mean ± SD	Follow-up median [IQR]
Gait	AvgSpeed (gait speed)	115	1.08 ± 0.22	1.06 [0.922–1.21]	97	1.05 ± 0.225	1.09 [0.886–1.21]
Gait	CycleVariability	115	0.211 ± 0.0284	0.207 [0.195–0.225]	97	0.212 ± 0.031	0.208 [0.193–0.224]
Gait	DoubleStance	115	0.248 ± 0.0388	0.246 [0.225–0.276]	97	0.245 ± 0.0359	0.247 [0.223–0.267]
Gait	MeanStepDuration	115	0.549 ± 0.05	0.55 [0.514–0.581]	97	0.555 ± 0.0497	0.551 [0.519–0.594]
Gait	RoliTronc	115	0.171 ± 0.0611	0.162 [0.121–0.212]	97	0.175 ± 0.0699	0.159 [0.123–0.216]
Balance	Balance score (YF)	127	89.7 ± 20.8	99 [91–99]	92	85.8 ± 23.8	98 [86.5–99]
Balance	Balance score (YO)	127	90.7 ± 19.2	99 [92–99]	92	88.9 ± 18	97 [86.8–99]
Balance	Surface (YF)	127	3.84e+04 ± 4.33e+05	1.63 [0.943–3.18]	92	4.1 ± 7.55	2.43 [1.25–3.9]
Balance	Surface (YO)	127	3.49e+04 ± 3.93e+05	1.31 [0.734–2.48]	92	2.33 ± 2.53	1.69 [0.959–2.84]
Balance	Sway density (YF)	127	28.9 ± 4.9	29.9 [29.8–29.9]	92	29.1 ± 3.17	29.8 [29.8–29.9]
Balance	Sway density (YO)	127	29.1 ± 4.13	29.9 [29.8–29.9]	92	29.6 ± 1.59	29.8 [29.8–29.9]

Instrumented measures are summarized at baseline and follow-up with sample size (n) and descriptive statistics (mean ± SD, and median [IQR]) for each measurement. 131 participants overall had at least one instrumented measure at baseline, 105 at follow-up and 105 at both time points. Sample sizes vary across parameters due to missing data. YO: eyes open; YF: eyes closed.

As detailed in Table 5, frailty prevalence estimates varied across definitions, with FRAGIRE self-reported status identifying 7% frail and 54% pre-frail (n = 123). Agreement between expert-based and Fried binary classifications was low to moderate and did not reach statistical significance (χ^2^ = 2.65, Cramér’s V = 0.16, p = 0.103; n = 105). Regarding score-based associations, Frailty_Score and Fried_Score were significantly correlated (Spearman’s ρ = −0.33, p < 0.001, Bonferroni-significant), while correlations with self-rated frailty (Auto_Evaluation) were weaker and not significant after correction (Frailty_Score vs. Auto_Evaluation: ρ = 0.22, p = 0.025; Fried_Score vs. Auto_Evaluation: ρ = −0.13, p = 0.195).

**TABLE 5:** Frailty prevalence, agreement between frailty definitions, correlations between frailty scores, and 6-month prospective falls incidence.

Domaine	Measure	Result	Sample size
Frailty prevalence	Expert frailty (Frailty_State)	26.7% frail	n = 105
	Fried frailty (Fried_State ≥3)	22.9% frail	n = 105
	FRAGIRE (self-report)	7% frail/54% pre-frail	n = 123
Agreement	Expert vs. Fried (binary)	χ^2^ = 2.65, Cramér’s V = 0.16, p = 0.103	n = 105
Correlations	Frailty_Score vs. Fried_Score	ρ = −0.33, p < 0.001 (Bonferroni-significant)	n = 105
	Frailty_Score vs. Auto_Evaluation	ρ = 0.22, p = 0.025 (NS after correction)	n = 105
	Fried_Score vs. Auto_Evaluation	ρ = −0.13, p = 0.195	n = 105
Falls incidence	Prospective falls (6 months)	6% (7 participants, 8 falls)	n = 107
	Falls vs. Fried_Score	Higher median Fried score in fallers (NS)	n = 105

Frailty prevalence was estimated using three definitions: expert-assessed frailty (Frailty_State), Fried phenotype (Fried_State ≥3), and FRAGIRE, self-reported frailty status. Agreement between expert and Fried binary classifications was assessed using Pearson’s chi-square test and Cramér’s V. Associations between frailty-related scores (Frailty_Score, Fried_Score, and Auto_Evaluation) were assessed using Spearman’s rank correlation coefficient (ρ). Bonferroni correction was applied for multiple correlation tests. Prospective falls were recorded during 6 months of follow-up; differences in Fried score between fallers and non-fallers were explored (non-significant). Sample sizes vary depending on available data.

Prospective falls over 6 months were infrequent, with seven participants reporting eight falls (6%) (n = 107). In cross-sectional comparisons, fallers tended to have a higher median Fried score than non-fallers, although this difference was not statistically significant, and the low number of fall events limited statistical power for fall-related analyses (Table 5).

#### 3.2.2 Relation between frailty measures

A significant positive correlation was found between expert-assigned Frailty_Score and patient self-evaluation (Auto_Evaluation) (rho = 0.22, p = 0.025) using conventional significance thresholds. However, this relationship does not remain significant after Bonferroni correction (p > 0.00333), suggesting a more delicate or discussable relationship between expert judgment and patients’ perception of their own health status. The correlation between Fried_Score and Auto_Evaluation was not statistically significant (rho = −0.13, p = 0.195), indicating that standardized medical criteria may not align well with patients’ self-perceived frailty status within our dataset.

**FIGURE 3:**
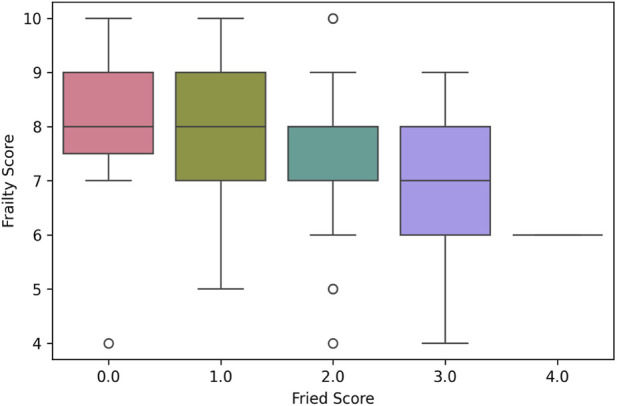
Distribution of expert-rated Frailty_Score (higher = less frail) across Fried_Score categories (higher = more frail) n = 105.

Spearman correlation analysis revealed a significant negative correlation between expert-assigned **Frailty_Score** and **Fried_Score** (rho = - 0.33, p < 0.001). This correlation remains significant even after Bonferroni correction (p < 0.00333). This negative relationship was expected due to the opposite scaling directions of these measures, with higher Frailty_Scores indicating better health and higher Fried_Scores indicating worse health. The moderate strength of this correlation suggests that while expert assessment and standardized criteria capture related aspects of frailty, they also assess distinct components (Figure 3).

##### 3.2.2.1 Agreement between binary frailty classifications

Chi-square analysis of binary classifications revealed a non-significant association between expert-determined frailty status (Frailty_State) and Fried criteria-based classification (Fried_State) (χ^2^ = 2.65, V = 0.16, p = 0.103).

##### 3.2.2.2 Comparison of continuous scores across binary classifications

Frailty_Score differed significantly between frail and non-frail groups as classified by Fried criteria (U = 1,318, p = 0.007, r = 0.27) using conventional significance levels. However, this relationship does not remain significant after Bonferroni correction (p > 0.00333). Fried_Score differed significantly between frail and non-frail groups as classified by expert assessment (U = 724, p = 0.007, r = 0.26) using conventional significance levels, but this relationship also loses significance after Bonferroni correction (p > 0.00333).

Supervised classification models trained only on instrumented balance and gait variables (forceplatform sway metrics, IMUderived gait parameters) showed limited ability to recover Fried defined binary frailty status (gold standard). The best performance obtained under this setting reached an Area Under the curve (AUC). Model performance improved substantially after the inclusion of clinical assessment and subjective variables with the best multimodal configuration achieving an AUC.

In the full multimodal model, several subjective/clinical measures—e.g., *FROPCOM item23* “During the observation of walking and turning, does the person appear unstable or at risk of losing balance? These findings indicated on one hand that objective static postural control and gait metrics alone were insufficient to approximate Fried frailty. On the other hand incorporating patient-reported and clinician-rated information was necessary to capture the multidimensional construct of geriatric frailty.

As shown in Table 6, functional performance assessed by SPPB9 and clinician-observed instability (FROPCOM0023) were examined across two age strata (65–74 vs. ≥ 75 years). Among participants with available SPPB9 data, 103 were aged 65–74 years and 32 were aged ≥75 years. The SPPB9 mean ± SD was 15.12 ± 5.09 in the 65–74 group and 16.91 ± 4.94 in the ≥75 group, with median [IQR] values of 14.73 [12.16–17.20] and 16.63 [13.17–19.43], respectively.

**TABLE 6:** Domain-level functional performance and clinician-observed instability across age strata (65–74 vs. ≥ 75 years).

AgeStrata	n (SPPB9)	SPPB9 mean ± SD	SPPB9 median [IQR] (s)	n (FROPCOM0023)	FROPCOM0023 (%positive)
65–74	103	15.12 ± 5.09	14.73 [12.16–17.20]	102	22.5
≥75	32	16.91 ± 4.94	16.63 [13.17–19.43]	31	38.7

- SPPB9 is reported in seconds (higher values indicate poorer performance).

- FROPCOM0023 is a binary clinician-rated item (“During the observation of walking and turning, does the person appear unstable or at risk of losing balance?“) reported as percentage of positive responses.

- Values are reported as mean ± SD and median [IQR].

- Sample sizes differ across variables due to missing data.

For clinician-observed instability, FROPCOM0023 data were available for 102 participants aged 65–74 and 31 participants aged ≥75. The proportion of positive responses was higher in the older group (38.7%) compared with the 65–74 group (22.5%), suggesting a greater frequency of clinician-observed gait instability with increasing age (Table 6).

SPPB9 is expressed in seconds (with higher values indicating poorer performance), while FROPCOM0023 represents the percentage of participants judged as unstable or at risk of losing balance during walking and turning. Sample sizes varied across measures due to missing data.

As shown in Table 7, functional performance assessed using the SPPB9 score differed across fall-risk groups defined by clinician-observed instability (FROPCOM0023). Among participants with available SPPB9 measurements, 27 were classified as high fall risk (HFR; FROPCOM23 = 1) and 101 as low fall risk (LFR; FROPCOM23 = 0). The mean SPPB9 score was higher in the HFR group (17.57 ± 4.22) compared with the LFR group (15.04 ± 5.30). Similarly, the median [IQR] SPPB9 was higher in HFR (17.23 [14.87–20.16]) than in LFR (13.79 [12.10–17.02]).

**TABLE 7:** Functional performance (SPPB9) across fall-risk groups defined by clinician-observed instability (FROPCOM0023).

Fall Risk Group	n (SPPB9)	SPPB9 mean ± SD	SPPB9 median [IQR] (s)
HFR (FROPCOM23 = 1)	27	17.57 ± 4.22	17.23 [14.87–20.16]
LFR (FROPCOM23 = 0)	101	15.04 ± 5.30	13.79 [12.10–17.02]

Sample sizes reflect available SPPB9 measurements within each group. HFR: High fall Risk. LFR: Low Fall Risk.

As shown in Table 8, frailty stages defined using the Fried phenotype were associated with an increasing psychological burden. Among participants with available Fried scores (n = 130), 11 (8.5%) were classified as robust, 87 (66.9%) as pre-frail, and 32 (24.6%) as frail.

**TABLE 8:** Frailty stages and psychological burden.

Fried stage	n (Fried)	n (HADS-A)	HADS anxiety ≥11 (%)	n (HADS-D)	HADS depression ≥11 (%)	n (SF-36 emotional)	SF-36 emotional wellbeing (mean ± SD)
Robust (0)	11	8	0.0	8	0.0	8	79.0 ± 12.2
Pre-frail (1,2)	87	78	19.2	78	3.8	78	63.8 ± 20.3
Frail (≥3)	32	26	50.0	26	19.2	26	55.4 ± 18.4

- Fried stages were defined using Fried_Score (0 = robust, 1–2 = pre-frail, ≥3 = frail).

- HADS, anxiety/depression are reported as the percentage of participants with pathological scores (≥11).

- SF-36, emotional wellbeing is reported as mean ± SD.

The proportion of participants with pathological HADS anxiety scores (≥11) increased across frailty stages, from 0.0% in robust participants (n = 8) to 19.2% in the pre-frail group (n = 78) and 50.0% in frail participants (n = 26). Similarly, pathological HADS depression scores (≥11) rose from 0.0% in robust participants (n = 8) to 3.8% in the pre-frail group (n = 78) and 19.2% in frail participants (n = 26).

In parallel, SF-36 emotional wellbeing decreased with increasing frailty severity, with mean ± SD values of 79.0 ± 12.2 in the robust group (n = 8), 63.8 ± 20.3 in the pre-frail group (n = 78), and 55.4 ± 18.4 in the frail group (n = 26) (Table Z). Sample sizes varied across measures due to missing data.

##### 3.2.2.3 Impact of physical activity, kinesitherapy and adapted physical activity on gait and balance.

To clarify how improving postural control and locomotion relate to fall risk, we compared older adults who were sedentary versus active (≈two to four h/wk of physical activity), who did versus did not receive kinesiotherapy, and who did versus did not participate in adaptive physical activity (APA). Instrumented gait (IMU) and balance (force platform) measures were analyzed to determine whether these exposures were associated with functional mobility and postural control.

Expert-assessed gait speed at baseline—without follow-up—revealed significant differences between participants engaged in regular physical activity (p = 0.0136) and those in kinesiotherapy (p = 0.0012).

At baseline, participants assigned to the kinesiotherapy group walked significantly more slowly than those in the comparison group (p = 0.034), consistent with preferential enrollment of individuals with greater mobility limitation into rehabilitation. By 6 months (M+6) the between-group difference had narrowed and was no longer statistically significant (p = 0.055), suggesting possible convergence. Within the kinesiotherapy group, gait speed showed no appreciable change from baseline to M+6 (p = 0.779), indicating no detectable intervention effect on this outcome over the study interval.

**FIGURE 4:**
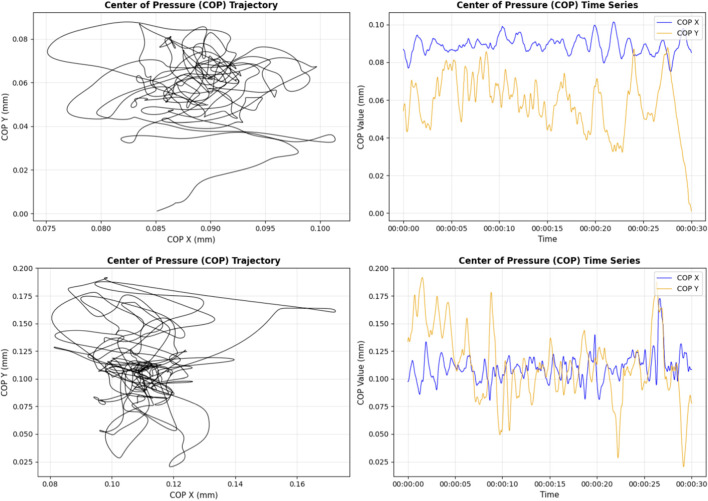
Examples of center-of-pressure (COP) trajectories for a robust participant (top) and a frail participant (bottom).

Further analysis of center of pressure (CoP) parameters highlighted the role of kinesiotherapy in static postural control (Figure 4). Under eyes-closed conditions, baseline CoP speed was significantly higher in the kinesiotherapy group (*p* = 0.0176), but this difference was no longer significant at M+6 (p = 0.171), possibly indicating normalization of postural function. The change in CoP speed over time was statistically significant (p = 0.017), suggesting an adaptive response to rehabilitation. In eyes-open conditions, CoP speed was significantly different at baseline (p = 0.0177), and while the difference at M+6 approached significance (p = 0.054), the overall change from baseline was significant (p = 0.0178). These results support the hypothesis that rehabilitation interventions contribute to improve postural stability.

Mediolateral (ML) CoP variation provided additional insights into balance mechanisms. Under eyes-closed conditions, ML variation was significantly different at both baseline (p = 0.0176) and M+6 (p = 0.0021), with a significant overall change over time (p = 0.0178). These findings suggest that kinesiotherapy effectively reduces lateral sway, contributing to improved ML postural control. A similar pattern was observed in eyes-open conditions, with significant differences at baseline (p = 0.0176) and M+6 (p = 0.0097), as well as a significant longitudinal change (p = 0.0177). Notably, participants engaging in general physical activity—independent of structured rehabilitation—also demonstrated significantly improved ML stability at M+6 (p = 0.0049), highlighting the contribution of regular physical activity to static postural control.

## 4 Discussion

The primary objective of the 5P-Échelle cohort was to evaluate the feasibility and preliminary performance of a portable, IoT-supported assessment platform combining IMU-based gait analysis, static balance assessment, grip strength, and standardized questionnaires for early frailty detection in an outpatient primary care setting. While the long-term ambition is to support partially demedicalized frailty screening pathways, the present analysis focused on baseline and early follow-up data collected by trained medical personnel. Overall, three central messages emerge from this work: **(**i) the feasibility and reliability of using an IoT-based platform for frailty detection in real-world primary care setting, (ii) the potential contribution of this platform to scalable and partly demedicalized screening workflows, and (iii) the opportunity to move toward individual longitudinal monitoring and, ultimately, digital-twin–oriented prevention strategies.

### 4.1 Feasibility and reliability of IoT-based frailty detection in primary care

Our results indicate that the assessment workflow is feasible and acceptable in ambulatory primary care settings. High completion rates across visits and follow-up contacts support operational compatibility with prevention-oriented care pathways and suggest that the platform can integrate sensor-based functional testing with standardized clinical and patient-reported measures without specialized infrastructure.

Beyond feasibility, the cohort profile highlights the relevance of early screening in primary care. Despite a relatively young-old sample, impairments across intrinsic capacity domains were frequent, supporting the need for tools that can detect vulnerability before overt disability emerges (Leung et al., 2022). In this respect, the platform is consistent with intrinsic capacity–oriented approaches such as the WHO ICOPE framework, which aims to promote early identification and prevention in scalable community-compatible pathways (Integrated care for older peoplea).

From a reliability perspective, the platform captured coherent gradients across clinically meaningful strata, particularly for fall-related risk markers. Stratified analyses across age groups and clinician-observed instability groups showed consistent patterns in functional performance and instability-related indicators, supporting the clinical interpretability of the digital outputs and their relevance for early risk profiling.

### 4.2 Multidimensional frailty constructs: Agreement, convergence, and complementarity

Most importantly, score-based analyses showed convergence between the Frailty Score and the Fried Score, as assessed by the platform, rated by experts. The moderate level of agreement suggests that there are overlapping yet non-redundant dimensions, which supports the idea that frailty assessment benefits from multimodal integration. Different frameworks may also provide complementary information in primary care contexts, where vulnerability often involves a combination of functional limitations, clinical judgement, and psychosocial factors.

However, agreement between binary frailty classifications based on expert judgement and the Fried phenotype did not reach statistical significance. This absence of strict concordance is to be expected, given the multidimensional nature of frailty and the conceptual differences between global clinical assessment and phenotype-based operational definitions. The Fried phenotype remains a widely adopted construct with predictive validity for adverse outcomes, but it primarily captures a specific physical aspect of frailty (Fried L. P. et al., 2001).

Self-rated frailty showed weak and unstable alignment with clinician-based and standardized measures, which reinforces the view that subjective perception should be interpreted as contextual information rather than as a screening strategy in its own right, particularly in the early stages.

### 4.3 What digital markers add—And why multimodal integration matters

Our predictive analyses provide two practical insights for digital frailty workflows. First, models based solely on instrumented gait and balance features showed limited ability to recover Fried-defined frailty status, suggesting that biomechanical signals alone cannot fully approximate a multidimensional construct involving fatigue, function, mood, and health perception. Second, performance improved substantially when clinical and patient-reported variables were integrated, resulting in the best discrimination in multimodal configurations.

The importance of performance-based measures and structured clinical observation is consistent with the role of tools such as the SPPB, which has demonstrated validity and reliability for functional assessment in older adults and remains an important bridge between objective measurement and clinical meaning (Gómez et al., 2013). Overall, these findings suggest that IoT-derived metrics are most useful when embedded into an integrative assessment framework rather than treated as standalone proxies for frailty.

### 4.4 Fall-related outcomes and sensitivity to functional change

The low incidence of falls during follow-up limited statistical power for fall-related predictive analyses. This limitation is common in cohorts with preserved functional status and relatively short follow-up, and it constrains conclusions regarding fall prediction performance. Nevertheless, prospective monitoring aligns with recommended methodological standards aimed at minimizing recall bias and improving outcome validity (Jehu and Skelton, 2024).

Despite limited fall events, the platform demonstrated clinically coherent fall-related patterns at baseline, including gradients in functional performance across clinician-observed instability strata. In addition, balance parameters appeared responsive to rehabilitation and physical activity exposure, suggesting potential sensitivity to short-term neuromuscular adaptations. This supports the use of repeated digital functional assessments as part of prevention-oriented monitoring strategies.

### 4.5 Demedicalization and scalability: From feasibility to implementation

Although the present study relied on trained medical personnel, the platform was designed to support partially demedicalized workflows. Its portability, limited infrastructure needs, and standardized protocol make it compatible with deployment by trained non-medical staff in real-world settings, which could increase scalability and reduce barriers to systematic frailty screening. Future implementation studies should therefore specifically assess inter-rater reliability, usability, and acceptability when the workflow is delivered outside strictly medical contexts.

### 4.6 Individual longitudinal monitoring and a tangible digital twin workflow

Beyond cross-sectional screening, the platform naturally supports individual longitudinal monitoring by enabling repeated measurement of gait, balance, grip strength, clinician-observed instability, and patient-reported outcomes. In a future digital-twin–oriented workflow, these repeated data streams could feed a personalized model updated at each visit: **(i)** sensor-derived measures (IMU gait speed and variability, postural sway metrics), **(ii)** clinical observations (e.g., FROP-COM instability), and **(iii)** questionnaire-based domains (functioning, mood/anxiety, intrinsic capacity screening). The system could then generate individualized feedback loops—such as alerts for deterioration, recommendations for targeted exercise or fall-prevention interventions, and prompts for clinical review—while subsequent assessments would refine predictions and track trajectories over time. Importantly, this digital twin perspective remains forward-looking in the present manuscript and will require dedicated methodological work (minimal feature selection, interpretability constraints, external validation, and implementation testing) before clinical translation.

### 4.7 Strengths and limitations

This pragmatic primary care study provides a multidimensional characterization of early frailty-related vulnerability by combining sensor-derived functional measures with clinician-rated observations and standardized questionnaires. Prospective falls were monitored using widely accepted methodological standards, although the low number of events limited fall-related analyses (Lamb et al., 2005). The study is also aligned with prevention-oriented intrinsic capacity frameworks that support scalable care pathways (Integrated care for older peopleb). Importantly, this work was enabled by a strong cross-institutional collaboration spanning engineering, primary care, geriatrics, and public health, which was essential for the development, deployment, and pragmatic evaluation of a complex digital health platform under real-world conditions.

Several limitations should be acknowledged. The participation rate was modest, which may have introduced selection bias toward healthier or more motivated individuals. In addition, follow-up duration was limited and falls were infrequent, restricting statistical power and the robustness of fall-related inferences. Finally, although the platform was designed to support deployment by trained non-medical staff in partially demedicalized workflows, this operational component was not directly evaluated in the present study and warrants dedicated implementation research.

## 5 Conclusion

This study provides preliminary evidence supporting the feasibility and clinical coherence of a portable, IoT-supported frailty assessment platform integrating sensor-derived gait and balance measures with grip strength and standardized clinical and self-reported indicators in primary care. Sensor-only models were insufficient to recover frailty status, whereas multimodal integration improved discrimination and highlighted the complementary roles of objective functional markers, clinician observation, and patient-reported outcomes. These results support further validation in larger cohorts, implementation studies involving non-medical personnel, and comparison with standard comprehensive geriatric assessment.

## Data Availability

The raw data supporting the conclusions of this article will be made available by the authors upon reasonable request, without undue reservation.
